# Common variants of *ZNF750*, *RPTOR* and *TRAF3IP2* genes and psoriasis risk

**DOI:** 10.1007/s00403-013-1407-9

**Published:** 2013-09-05

**Authors:** T. Dębniak, E. Soczawa, M. Boer, M. Różewicka-Czabańska, J. Wiśniewska, P. Serrano-Fernandez, A. Mirecka, K. Paszkowska-Szczur, J. Lubinski, L. Krysztoforska, Z. Adamski, R. Maleszka

**Affiliations:** 1 Department of Genetics and Pathology, International Hereditary Cancer Center, Pomeranian Medical University, Połabska 4, 70-115 Szczecin, Poland; 2 Department of Dermatology and Venerology, Pomeranian Medical University, Szczecin, Poland; 3 Regional Hospital, Koszalin, Poland; 4 Clinic of Dermatology and Venerology, Poznan University of Medical Science, Szczecin, Poland

**Keywords:** Psoriasis vulgaris, *TRAF3IP2*, *ZNF750*, *RPTOR*

## Abstract

Psoriasis vulgaris is a genetically heterogenous disease with unclear molecular background. We assessed the association of psoriasis and its main clinical phenotypes with common variants of three potential psoriasis susceptibility genes: *ZNF750*, *RPTOR* and *TRAF31P2*. We genotyped 10 common variants in a cohort of 1,034 case–control individuals using Taqman genotyping assays and sequencing. Minor alleles of all four *TRAF3IP2* variants were more frequent among cases. The strongest, significant association was observed for rs33980500 (OR = 2.5, *p* = 0.01790). Minor allele of this SNP was always present in two haplotypes found to be associated with increased psoriasis risk: rs13196377_G + rs13190932_G + rs33980500_T + rs13210247_A (OR = 2.7, *p* = 0.0054) and rs13196377_A + rs13190932_A + rs33980500_T + rs13210247_G (OR = 1.8, *p* = 0.0008). Analyses of clinically relevant phenotypes revealed association of rs33980500 with pustular psoriasis (OR = 1.2, *p* = 0.0109). We observed significant connection of severity of cutaneous disease with variation at rs13190932 and suggestive with three remaining *TRAF3IP2* SNPs. Another positive associations were found between age of onset and familial aggregation of disease: smoking and younger age of onset, smoking and occurrence of pustular psoriasis, nail involvement and arthropatic psoriasis, nail involvement and more severe course of psoriasis. We found no statistically significant differences in the prevalence of the examined variants of *RPTOR* and *ZNF750* genes among our cases and controls. We have replicated the association of TRAF3IP2-_rs33980500 variant with the susceptibility to psoriasis. We have found new associations with clinically relevant subphenotypes such as pustular psoriasis or moderate-to-severe cases. We ascertain no connection of *RPTOR* and *ZNF750* variants with psoriasis or its subphenotypes.

**Electronic supplementary material:**

The online version of this article (doi:10.1007/s00403-013-1407-9) contains supplementary material, which is available to authorized users.

## Introduction

Psoriasis is one of the most common skin disorders. It is estimated that it affects 2–3 % of general Caucasian population [19]. Psoriasis is a chronic inflammatory skin disorder characterized by keratinocyte hyperproliferation and cutaneous increased blood flow induced by the stimulation of tissue resident immune cells and the marked alteration of cytokine profiles [1]. Current evidence suggests that psoriasis is an immune-mediated disorder and novel therapies involved in the suppression of the immune responses, such as the T cell-targeted agents and tumor necrosis factor (TNF) inhibitors, have improved the outcome of the disease [9, 18]. However, not all patients respond to these therapies and the efficacy varies between psoriasis patients. As judged by twin- and large population-based genetic studies, the disease seems to have a strong genetic component—disease concordance in monozygotic twin pairs amounts to at most 70 % and the sibling recurrence risk of psoriasis vulgaris (PsV) has been estimated to range between 4 and 11 [11]. To date, molecular background of this disease remains unclear [6]. At present, it is generally accepted that the disease is both multifactorial and genetically heterogeneous. Recently, three GWAS studies identified 6q21 as a new psoriasis susceptibility locus and suggested a possible association of *TRAF31P2* gene (OMIM 607043) with this disease [7, 8, 13]. Consistently, recent study of Spanish patients implicated genetic variants of this gene in psoriasis vulgaris development [17]. TRAF3IP2 protein is involved in inflammatory pathways, including cytokine signaling. To date, the gene has not been studied in Slavic population. Part of the genetic susceptibility of the disease could also be explained by demonstrated linkage or association of familial psoriasis to a locus on 17q25 (PSORS2) [4, 14, 26]. Recent studies indicate that mutations/polymorphisms within *CARD14* gene, located within this locus, predispose to psoriasis [15, 16]. However, it is possibly that this region contains more psoriasis susceptibility genes. Literature data point at two such genes: *ZNF750* (OMIM 610226) and *RPTOR* (OMIM 607130), also located on 17q25. The zinc-finger 750 (ZNF750) protein is a transcription factor involved in epidermis differentiation, required for terminal epidermal differentiation. Germline mutations cosegregating with the disease were found among familial cases; one mutation was also present in sporadic psoriasis patient but absent in healthy controls [28]. Regulatory-associated protein of mTOR (RAPTOR) regulates cell growth and survival. There are two reports suggesting an association between *RPTOR* polymorphisms and psoriasis [4, 10] and one indicating that such association does not exist [24]. Due to paucity and partial divergence of the *ZNF750* and *RPTOR* literature data, it is justified to perform population-based association study to evaluate possible link between selected mutations/polymorphisms of these genes and psoriasis. Herein, we genotyped ten common variants of *ZNF750*, *Rptor* and *TRAF31P2* genes in our case–control cohort (*n* = 1034) from Polish population and evaluated main clinical phenotypes of psoriasis. No such study has been performed up to now.

## Patients and methods

### Patients

The case group consisted of 517 (218 women and 299 men, mean age 42.9) unselected, consecutive patients with PsV from north-western Poland. Patients were recruited between 2010 and 2012 from the outpatients’ clinics and hospital wards of the two participating dermatology departments: (1) Chair and Clinic of Skin and Veneral Diseases, PMU, Szczecin; (2) Clinic of Dermatology and Venerology, PUMS, Poznań. Participation rates were over 80 % for both centers. All patients were at least 18 years old, although the disease could have been diagnosed at an earlier age.

Each patient was examined by dermatologist to evaluate disease type and severity. Psoriasis Area Severity Index (PASI) and Nail Psoriasis Severity Index (NAPSI) scores were used. Individuals with PASI index from 1 to 10 were regarded as mild disease group, cases with PASI >10 as moderate-to-severe group. Individuals with NAPSI =0 were regarded as patients without nail involvement, while patients with NAPSI =1 or more were defined as cases with nail disease.

A detailed questionnaire concerning age of diagnosis, family history of psoriasis, smoking, personal cancer history and cancer family history was collected.

Controls consisted of 517 healthy adults, who were sex- and age-matched with the cases. All controls came from the West Pomerania region of Poland. Control samples were selected from a population-based study of 1.5 million individuals from West Pomeranian who were enrolled in a study aimed at identifying familial aggregations of malignancies performed recently by our center. Individuals with psoriasis were excluded from the control group.

The study conformed with the Declaration of Helsinki and all participants signed an informed consent document prior to donating a blood sample. The study was approved by the institutional review board of the Pomeranian Medical University.

### Methods

DNA samples were obtained from peripheral blood of individuals. We analyzed three common *ZNF750* variants (rs8074277, rs11077947, rs12450046), three common *RPTOR* changes (rs11658698, rs12602885, rs869190) and four common variants of *TRAF3IP2* (rs33980500, rs13210247, rs13190932, rs13196377). All SNPs were analyzed by real-time PCR, using the LightCycler480 from Roche. The analyses were performed using TaqMan^®^ genotyping assay, consisting of sequence-specific primers and oligonucleotide fluorescent-labeled probes, which enabled amplification of examined fragments and further allele discrimination. Randomly selected probes were sequenced to confirm the results of real-time PCR

### Statistical methods

The first goal was to determine which factors, under those analyzed, may affect the disease risk considering affectedness and censoring age. For that aim, healthy controls and diseased cases were censored for age at last contact and age at diagnosis, respectively. The analysis was performed using a Cox regression, stratified by year of birth and sex.

The second goal was to establish how genetic and clinical factors could influence each other. In this case, only diseased subjects were taken into consideration. Factors affecting a binary-dependent variable—joints involvement, nails involvement, family history of psoriasis, severity of cutaneous affection—were analyzed with the help of a multivariate logistic regression model. In standard clinical practice, the severity of cutaneous affection is categorized into mild (PASI ≤10) or moderate-to-severe psoriasis (PASI >10) for simplicity [15]. We followed the same rule to keep the analysis as simple as possible. In contrast, there was one situation with a quantitative-dependent variable (age at diagnosis), whereas all independent factors were qualitative. For this situation, a multivariate analysis of variance was used instead.

Both the Cox regression, the logistic regression, and the analysis of variance used are multivariate models (i.e., just one model with multiple genetic predictors); corrections for multiple testing are thus intrinsic to the model. However, for the second goal (see above), there were not only several independent variables, but also several dependent variables: 4 binary (joints involvement, nails involvement, family history of psoriasis and severity of cutaneous affection) and 1 discrete (age at diagnosis). Thus, Bonferroni correction for multiple testing was applied for 5 converging tests.

Estimation of haplotype frequencies and their potential association with the disease risk were performed using the haplo.stats CRAN package (version 1.6.3) by Sinnwell and Schaid for R [23]. Linkage disequilibrium between SNPs for a given haplotype was calculated using the software JLIN by Carter et al. [5]. All statistical analyses were performed using the R software environment (version 2.15.2) [21].

## Results

### Association with disease

Comparing 517 cases against 517 controls, we found significant association (both by allele and by genotype) of TRAF3IP2_rs33980500 with increased psoriasis risk (Tables 1, 2). Heterozygous carriers of risk allele (rs33980500_CT genotype) had OR = 2.4, 95 % CI 1.07–5.17, *p* = 0.0325); homozygous mutation carriers were too rare to reach statistical power (11 cases vs. 3 controls). Individuals carrying one or two copies of the TRAF3IP2_rs33980500 risk allele were significantly overrepresented among patients (OR = 2.5, 95 % CI 1.2–5.4, *p* = 0.0179).

**Table 1:** The prevalence of the common ZNF750, RAPTOR and TRAF3IP2 variant genotypes

Gene	SNP	Genotype	Cases	Controls	OR	95 % CI	*p*
RAPTOR	rs869190	GG	311 (60.15 %)	296 (57.36 %)	–	–	–
		GT	169 (32.68 %)	168 (32.55 %)	0.961	0.553–1.671	0.890
		TT	28 (5.41 %)	24 (4.65 %)	1.288	0.424–3.906	0.654
	rs11658698	CC	249 (48.16 %)	256 (49.61 %)	–	–	–
		CT	223 (43.13 %)	198 (38.37 %)	1.204	0.887–1.635	0.233
		TT	40 (7.70 %)	49 (9.40 %)	0.899	0.522–1.549	0.701
	rs12602885	GG	328 (63.40 %)	314 (60.80 %)	–	–	–
		AG	164 (31.70 %)	165 (31.90 %)	0.961	0.549–1.680	0.889
		AA	22 (4.25 %)	21 (4.06 %)	1.040	0.314–3.440	0.948
ZNF750	rs8074277	TT	287 (55.50 %)	291 (56.39 %)	–	–	–
		CT	201 (38.80 %)	184 (35.65 %)	1.083	0.061–19.008	0.956
		CC	24 (4.60 %)	30 (5.80 %)	NA	NA (0 to infinity)	NA
	rs11077947	AA	120 (23.20 %)	132 (25.60 %)	–	–	–
		AG	260 (50.20 %)	245 (47.48 %)	0.993	0.670–1.473	0.975
		GG	134 (25.90 %)	118 (22.80 %)	1.093	0.672–1.776	0.719
	rs12450046	GG	284 (54.90 %)	284 (55.03 %)	–	–	–
		AG	198 (38.29 %)	179 (34.68 %)	0.8860	0.051–15.241	0.933
		AA	24 (4.60 %)	30 (5.80 %)	NA	NA (0 to infinity)	NA
TRAF3IP2	rs13190932	GG	408 (78.90 %)	430 (83.30 %)	–	–	–
		AG	93 (17.98 %)	60 (11.60 %)	1.107	0.158–7.724	0.918
		AA	10 (1.90 %)	2 (0.38 %)	NA	NA (0 to infinity)	NA
	rs13196377	GG	407 (78.70 %	432 (83.70 %)	–	–	–
		AG	86 (16.60 %)	58 (11.24 %)	0.451	0.073–2.767	0.389
		AA	10 (1.90 %)	2 (0.38 %)	NA	NA (0 to infinity)	NA
	rs13210247	AA	400 (77.36 %)	427 (82.75 %)	–	–	–
		AG	100 (19.30 %)	64 (12.40 %)	1.488	0.603–3.670	0.388
		GG	10 (1.90 %)	2 (0.38 %)	2.957	0.099–87.863	0.531
	rs33980500	CC	378 (73.11 %)	417 (80.80 %)	–	–	–
		**CT**	116 (22.40 %)	69 (13.40 %)	2.355	1.074–5.166	0.032*
		TT	11 (2.12 %)	3 (0.60 %)	NA	NA (0 to infinity)	NA

Asterisk indicates statistically significant (*p* < 0.05)

**Table 2:** The ZNF750, RAPTOR and TRAF3IP2 allele frequencies in PSV patients and healthy controls

Gene	SNP	Allele	OR	95 % CI	*p* value
RAPTOR	rs869190	G	–	–	–
		T	1.028206	0.64311–1.644	0.9075
	rs11658698	C	–	–	–
		T	1.053000	0.83505–1.328	0.6625
	rs12602885	G	–	–	–
		A	0.997476	0.61360–1.622	0.9919
ZNF750	rs8074277	T	–	–	–
		C	0.355599	0.04877–2.593	0.3077
	rs11077947	A	–	–	–
		G	1.058406	0.83402–1.343	0.6405
	rs12450046	G	–	–	–
		A	2.651415	0.37028–18.986	0.3316
TRAF3IP2	rs13190932	G	–	–	–
		A	1.187910	0.17743–7.953	0.8591
	rs13196377	G	–	–	–
		A	0.398488	0.06985–2.273	0.3004
	rs13210247	A	–	–	–
		G	1.527635	0.64806–3.601	0.3328
	rs33980500	C	–	–	–
		T	2.524526	1.17285–5.434	0.0179*

Asterisk indicates statistically significant (*p* < 0.05)

The incidence of the remaining common variants of *TRAF3IP2* gene was also increased among patients; however, the differences were statistically not significant (Tables 1, 2).

We found no statistically significant differences in the prevalence of the examined variants of *RPTOR* and *ZNF750* genes among cases and controls (Tables 1, 2)

### Association with age of onset

We found no statistically significant association between any of the ten examined variants (neither by allele, nor by genotype) and an earlier or later onset of the disease. Furthermore, there were no major differences in the mean age of diagnosis of PsV patients in subjects that were either homo- or heterozygous for any of the SNPs (data available on request).

We observed statistically significant association of smoking and familial aggregation of disease with age of psoriasis onset. Smokers had an average age at onset almost 2 years earlier than non-smokers (35.6 vs. 37.3; *p* = 0.024). Individuals with positive family history had an average age at onset almost 4 years earlier than sporadic cases (33.5 vs. 37.4; *p* = 0.0113).

### Association with disease severity

Carriers of TRAF3IP2_rs13190932 risk allele have increased risk of severe psoriasis (OR = 2.7, 95 % CI 1.2–5.8, *p* = 0.01266). Similarly, more severe course of disease seems to be associated with nail involvement (OR = 1.3, 95 % CI 1.08–1.44, *p* = 0.003). There was non-significant, suggestive association of the remaining *TRAF31P2* SNPs with more severe PASI (as shown in Online Resource 1—statistical analyses of clinical subphenotypes).

### Association with nail involvement

The distribution of all examined variants was similar in cases with and without nail psoriasis (as shown in Online Resource 1). We found significant overrepresentation of individuals with arthropatic psoriasis in the subgroup of patients with nail involvement (OR = 1.3, 95 % CI 1.1–1.5, *p* = 0.0388). Similar association was found for moderate-to-severe cases (OR = 1.01, 95 % CI 1.002–1.02 *p* = 0.00620).

### Association with joints involvement

Evaluation of arthropatic psoriasis as dependent variable revealed only one significant association—patients with nail involvement have increased risk of arthropatic psoriasis (OR = 1.2, 95 % CI 1.07–1.34, *p* = 0.00246) (please see Online Resource 1).

### Association with familial occurrence of the disease

We did not find any significant difference in the distribution of any of the examined genotypes among sporadic cases and individuals with 1st or 2nd degree relatives affected with psoriasis (data available on request).

### Association with psoriasis skin subtypes

Individuals carrying one or two copies of the TRAF3IP2_rs33980500 risk allele were significantly overrepresented among patients with pustular psoriasis (OR = 1.2, 95 % CI 1.04–1.34, *p* = 0.0109). Similar association was observed for smokers (OR = 1.1, 95 % CI 1.01–1.14, *p* = 0.0274).

### Haplotype frequency

#### TRAF3IP2

Haplotype analysis of *TRAF3IP2* gene revealed three major haplotype blocs that were present in 98 % of our cases (Table 3). The reference haplotype rs13196377_G + rs13190932_G + rs33980500_C + rs13210247_A was present in 85 % of cases and 91 % of controls.

**Table 3:** Haplotype frequency of examined TRAF3IP2 variants

rs13196377	rs13190932	rs33980500	rs13210247	*p* value	OR	95 % CI
G	G	C	A	–	–	–
A	A	T	G	0.0008	1.81	1.302–2.50
G	G	T	A	0.0054	2.74	1.387–5.43

Haplotypes not frequent enough to allow haplotype analysis were excluded from the table. The reference haplotype corresponds to the most frequent one (GGCA)

The remaining two haplotypes were significantly overrepresented among cases when compared to controls: (a) haplotype rs13196377_A + rs13190932_A + rs33980500_T + rs13210247_G found in 10 % of patients and 6 % of healthy individuals; (b) haplotype rs13196377_G + rs13190932_G + rs33980500_T + rs13210247_A present in 3 % of cases and 1 % of controls (Table 3).

#### RPTOR

There was no significant difference for any of the *RAPTOR* haplotypes among cases and healthy adults (as shown in Online Resource 2).

#### ZNF750

There was no significant difference for any of the *ZNF750* haplotypes among cases and healthy adults (as shown in Online Resource 3).

#### Linkage disequilibrium

All four *TRAF3IP2* SNPs are in linkage disequilibrium with each another (LR-Test, 1,000 iterations, *p* < 0.0001) (*R* square = 0.88; *D*′ = 0.96).

All three *RPTOR* variants are linkage disequilibrium with each another (LR-Test, 1,000 iterations, *p* < 0.0001) (*R* square = 0.89; *D*′ = 0.94).

All three *ZNF750* changes are linkage disequilibrium with each another (LR-Test, 1,000 iterations, *p* < 0.0001) (*R* square = 0.85; *D*′ = 0.92).

## Discussion

Psoriasis vulgaris is a heterogeneous disease that has a complex genetic background. Results of our multivariate and haplotype analyses point at association of *TRAF3IP2* gene with a disease. Minor alleles of all four variants were more frequent among cases. The strongest, significant linkage was observed for rs33980500 (OR = 2.5, *p* = 0.01790). It is the only SNP with minor allele present in all two risk haplotypes (rs13196377_G + rs13190932_G + rs33980500_T + rs13210247_A and rs13196377_A + rs13190932_A + rs33980500_T + rs13210247_G), and major allele always present in reference haplotype block. Thus, for the given population, it is enough to genotype TRAF3IP2_rs33980500 and the presence of at least one “T” allele would be associated with an ~twofold increased diseased risk.

Rs33980500 causes a mutation Asp19Asn in the protein sequence and the resulting change in charge (a negative electric charge to nonpolar) might have an impact on the protein structure and, hence, its function. Additionally, rs33980500 has recently been reported to encode a mutant protein with an almost completely disrupted binding property to TRAF6, supporting its impact as a main disease-causing variant and modulator of IL-17 signaling [3].

Rs13190932 is also a missense alteration (Arg74Trp) in the protein sequence (a positive electric charge changed to neutral). Two remaining variants (rs13196377 and rs13210247) are intronic alterations that, according to our results, are in linkage disequilibrium with coding variants studied herein.

To date, only a few studies evaluating *TRAF31P2* gene and psoriasis vulgaris have been published. In a recent GWAS study, two intronic variants (rs13210247, rs13196377) as well as one coding variant (rs13190932, p.R74W [NM_147686]) were convincingly associated with odds ratios (ORs) of up to 1.8. Additional sequencing revealed coding variant p.D19N (rs33980500) as strongly associated (*p* = 1.13 × 10 − 20, OR = 1.95) and the only variant present on all risk haplotypes [13]. Ellinghaus et al. reported an association of rs13210247 and rs33980500 with ORs 1.3–1.7, respectively. Rs 13190932, however, was found not to be associated with psoriasis [7]. In another GWAS study, significant association with psoriasis was found for two different *TRAF31P2* variants: rs240943 and rs458017 [8]. Finally, Julia et al. [17] replicated only one common variant, rs458017 and found a positive association of this SNP with disease.

A recent meta-analysis shows that rs33980500 is associated with the disease in all studied populations [27] and our results support this thesis. Due to genetic heterogeneity of European populations (marked for example by different proportions and frequencies of *BRCA1* mutations among German, Polish or Finnish individuals), it cannot be excluded that association power of *TRAF3IP2* with psoriasis is modified by many factors and may vary among different populations—as indicated by statistical values described in our Polish series (OR = 2.5, *p* = 0.0179), German Kiel cohorts (OR = 1.6, *p* = 5.8e−05, or CASP GWAS dataset (OR = 1.4, *p* = 3.3 × 10^−4^) [27].

Functional studies suggest *TRAF3IP2* gene as important factor in psoriasis development.

The protein product of *TRAF3IP2* gene, Act 1, binds with interleukin 17 receptor and is essential for IL17-dependent signaling in autoimmune and inflammatory disease, including psoriasis [12, 20]. IL-17 is expressed by activated T cells and upregulates pro-inflammatory cytokines, chemokines, and tissue-degrading matrix-metalloproteases, such as nuclear factor kappa-B (NFKB), IL6, granulocyte macrophage colony-stimulating factor (GMCSF), prostaglandin E2, neutrophil-mobilizing cytokines, intercellular adhesion molecule-1 (ICAM1) [25]. Additionally, Act1 binding with IL17 receptor allows the incorporation of TNF receptor-associated factors TRAF3 and TRAF6 into the signaling complex and thus activation of the MAPK-pathway [22]. Mitogen-activated protein kinases (MAPKs) are key components in various cellular signal transduction pathways that affect growth factor-induced proliferation, gene expression, and compensation for environmental changes.

In summary, results of both functional and association studies, endorsed by our current findings, strongly suggest *TRAF31P2* gene as psoriasis susceptibility gene.

Analyses of clinically relevant phenotypes revealed association of rs33980500 with pustular psoriasis. We also observed significant linkage of severity of cutaneous disease with variation at rs13190932 and suggestive with three remaining SNPs. We found no significant association of *TRAF3IP2* variants with age of onset, familial occurrence of the disorder, involvement of joints and nails. However, due to limited statistical power of our study, caused by small number of cases, type 1 statistical error cannot be excluded.

Another positive associations were found between age of onset and familial aggregation of disease: smoking and younger age of onset, smoking and occurrence of pustular psoriasis, nail involvement and arthropatic psoriasis, nail involvement and more severe course of psoriasis vulgaris.

Our results do not support the literature data pointing at *RPTOR* gene as associated with psoriasis development. The intronic variant rs869190 was identified by GWAS study to be significantly associated with psoriasis [10] and reported to be overrepresented in cases with a documented family history of psoriasis [4]. The two remaining SNPs, rs11658698 present in the promoter sequence and rs12602885 localized in the 5′UTR, have not been examined up to now. None of the three *RPTOR* changes were found to be overrepresented among our cases.

To our knowledge, within the *ZNF750* loci, we provide second study of common variants of this gene and their association with disease. Birnbaum et al. [2] sequenced the promoter and exon regions of *ZNF750* in 716 Caucasian psoriasis cases and 397 Caucasian controls. No individual variants were found to associate with psoriasis. Authors observed a nominal association between rare variants in the 5′ regulatory region of *ZNF750* and psoriasis; however, these variants did not segregate with the psoriasis phenotype within families [2]. We genotyped two SNPs localized within intron 1 and exon 2 boundary (rs11077947 and rs12450046) and one coding variant (rs8074277) responsible for Met235Val substitution. We found no significant differences in alleles distribution (including haplotypes) in our case–control cohort.

Study results do not add evidence of *RPTOR* and *ZNF750* as psoriasis susceptibility genes. It seems that, at least so far, *CARD 14* remains the only psoriasis susceptibility gene located within PSORS2 locus. Reasonable sample size with cases and controls and relatively high frequency of occurrence of genotyped variants excludes type 2 statistical error. However, selection of patients into smaller subgroups according to their clinical subphenotypes increases the risk of inadequate statistical power (for weak associations). Thus, our evaluation of clinical subphenotypes, which did not reveal any significant association of *RPTOR* and *ZNF750* common variants with any of the studied phenotypes, needs to be verified by additional studies performed on larger numbers of patients.

In conclusion, using a Polish case–control cohort of 1034 individuals, we have replicated the association of TRAF3IP2-_rs33980500 variant with the susceptibility to PsV. Additionally, we have found new associations with clinically relevant subphenotypes such as pustular psoriasis or moderate-to-severe cases (PASI). We ascertain no connection of *RPTOR* and *ZNF750* variants with psoriasis. Additional, large multi-center association studies have to be performed to confirm our findings and to evaluate a potential impact of the molecular status of the *TRAF31P2* gene on the treatment outcome.

## Electronic supplementary material

Below is the link to the electronic supplementary material.

Supplementary material 1 (PDF 136 kb)

Supplementary material 2 (PDF 62 kb)

Supplementary material 3 (PDF 126 kb)
