# Supplementary material for: Common variants of ZNF750, RPTOR and TRAF3IP2 genes and psoriasis risk
Source: Arch Dermatol Res. 2013 Sep 5;306(3):231–8. doi: 10.1007/s00403-013-1407-9 (PMC3955134; doi:10.1007/s00403-013-1407-9)
Supplement: Supplementary file 1 — Supplementary material 1 (PDF 136 kb) [file 403_2013_1407_MOESM1_ESM.pdf]

Onlime resource 1

Statistical analyses of clinical subphenotypes

```
******* Arthropatic psoriasis as dependent variable ***********

summary(glm(LZS~., data=CASES_PSO2))

glm(formula = LZS ~ ., data = CASES_PSO2)

Deviance Residuals:
     Min        1Q    Median        3Q       Max
-0.42311  -0.20327  -0.11464   0.01502   0.96786

Coefficients: (4 not defined because of singularities)

                      Estimate Std. Error t value Pr(>|t|)
sexM                 -0.066845   0.054074  -1.236  0.21788
FH01                 -0.022941   0.052442  -0.437  0.66226
yob                   0.001729   0.001668   1.037  0.30102
PASI                 -0.019932   0.057717  -0.345  0.73020
NAPSI                 0.195172   0.060400   3.231  0.00145 **
skinII                0.097059   0.380804   0.255  0.79908
skinIII               0.159373   0.383855   0.415  0.67846
skinIII/IV            0.001204   0.466529   0.003  0.99794
skinIV               -0.010698   0.431447  -0.025  0.98024
smoke                -0.067225   0.053928  -1.247  0.21404
pustular.            -0.156592   0.114603  -1.366  0.17338
RAPTOR_rs869190GT     0.243525   0.094650   2.573  0.01082
RAPTOR_rs869190TT     0.032131   0.384023   0.084  0.93340
RAPTOR_rs11658698CT   0.077493   0.054882   1.412  0.15954
RAPTOR_rs11658698TT   0.037785   0.095009   0.398  0.69129
RAPTOR_rs12602885AA   0.126285   0.400032   0.316  0.75258
RAPTOR_rs12602885AG  -0.176429   0.097033  -1.818  0.07056 .
ZNF750_rs8074277CC    0.276685   0.429038   0.645  0.51975
ZNF750_rs8074277CT    0.066472   0.063131   1.053  0.29367
ZNF750_rs11077947AG  -0.097620   0.068073  -1.434  0.15315
ZNF750_rs11077947GG  -0.064306   0.088665  -0.725  0.46915
ZNF750_rs12450046AA  -0.123881   0.377538  -0.328  0.74317
ZNF750_rs12450046AG         NA         NA      NA       NA
TRAF3IP2_rs13190932AA -0.035034   0.172259  -0.203  0.83905
TRAF3IP2_rs13190932AG  0.135438   0.327971   0.413  0.68009
TRAF3IP2_rs13196377AA        NA         NA      NA       NA
TRAF3IP2_rs13196377AG -0.020091   0.272795  -0.074  0.94137
TRAF3IP2_rs13210247AG -0.035376   0.148754  -0.238  0.81227
TRAF3IP2_rs13210247GG        NA         NA      NA       NA
TRAF3IP2_rs33980500CT -0.091284   0.105511  -0.865  0.38801
TRAF3IP2_rs33980500TT        NA         NA      NA       NA

                       OR          2.5 %    97.5 %
sexM                 0.93534051 8.412821e-01  1.039915
FH01                 0.97731972 8.818560e-01  1.083118
yob                  1.00173072 9.984621e-01  1.005010
PASI                 0.98026489 8.754167e-01  1.097671
NAPSI                1.21552000 1.079816e+00  1.368278
skinII               1.10192533 5.224095e-01  2.324306
skinIII              1.17277582 5.526841e-01  2.488588
skinIII/IV           1.00120492 4.012475e-01  2.498237
skinIV               0.98935898 4.247231e-01  2.304634
```

```
smoke                 0.93498502 8.412036e-01  1.039222
pustular.             0.85505279 6.830341e-01  1.070393
RAPTOR_rs869190GT     1.27573811 1.059731e+00  1.535774
RAPTOR_rs869190TT     1.03265291 4.864892e-01  2.191975
RAPTOR_rs11658698CT   1.08057496 9.703739e-01  1.203291
RAPTOR_rs11658698TT   1.03850743 8.620605e-01  1.251070
RAPTOR_rs12602885AA   1.13460523 5.180086e-01  2.485150
RAPTOR_rs12602885AG   0.83825833 6.930795e-01  1.013848
ZNF750_rs8074277CC    1.31875135 5.688077e-01  3.057457
ZNF750_rs8074277CT    1.06873138 9.443457e-01  1.209501
ZNF750_rs11077947AG   0.90699389 7.937082e-01  1.036449
ZNF750_rs11077947GG   0.93771793 7.881351e-01  1.115691
ZNF750_rs12450046AA   0.88348535 4.215399e-01  1.851655
ZNF750_rs12450046AG           NA           NA        NA
TRAF3IP2_rs13190932AA 0.96557291 6.889032e-01  1.353356
TRAF3IP2_rs13190932AG 1.14503832 6.020749e-01  2.177657
TRAF3IP2_rs13196377AA         NA           NA        NA
TRAF3IP2_rs13196377AG 0.98010971 5.742103e-01  1.672932
TRAF3IP2_rs13210247AG 0.96524286 7.211359e-01  1.291981
TRAF3IP2_rs13210247GG         NA           NA        NA
TRAF3IP2_rs33980500CT 0.91275818 7.422406e-01  1.122449
TRAF3IP2_rs33980500TT         NA           NA        NA
```

per allele:

```
a <- (glm(LZS~., data=CASES_PSO))
summary(a)
```

```
Coefficients:
                     Estimate Std. Error t value Pr(>|t|)
sexM                -0.077788   0.053196  -1.462  0.14522
FH01                -0.026037   0.051371  -0.507  0.61282
yob                  0.001600   0.001631   0.981  0.32768
PASI                -0.017683   0.056933  -0.311  0.75643
NAPSI                0.183749   0.059528   3.087  0.00231 **
skinII               0.063412   0.376746   0.168  0.86650
skinIII              0.122717   0.379436   0.323  0.74671
skinIII/IV          -0.053940   0.460297  -0.117  0.90683
skinIV              -0.043423   0.425609  -0.102  0.91884
smoke               -0.065502   0.053357  -1.228  0.22102
pustular.           -0.154118   0.113476  -1.358  0.17593
RAPTOR_rs869190      0.217056   0.088570   2.451  0.01511
RAPTOR_rs11658698    0.046144   0.040744   1.133  0.25875
RAPTOR_rs12602885   -0.153715   0.090753  -1.694  0.09185 .
ZNF750_rs8074277     0.195074   0.381208   0.512  0.60940
ZNF750_rs11077947   -0.027263   0.043434  -0.628  0.53092
ZNF750_rs12450046   -0.131905   0.372977  -0.354  0.72397
TRAF3IP2_rs13190932  0.113787   0.322549   0.353  0.72463
TRAF3IP2_rs13196377 -0.019017   0.268843  -0.071  0.94368
TRAF3IP2_rs13210247 -0.032103   0.144427  -0.222  0.82432
TRAF3IP2_rs33980500 -0.079622   0.104129  -0.765  0.44537
```

```
                      OR          2.5 %      97.5 %
sexM                0.92516077 8.335598e-01  1.026828
FH01                0.97429931 8.809778e-01  1.077506
yob                 1.00160125 9.984052e-01  1.004808
PASI                0.98247250 8.787369e-01  1.098454
NAPSI               1.20171410 1.069377e+00  1.350427
skinII              1.06546588 5.091580e-01  2.229598
skinIII             1.13056404 5.374255e-01  2.378330
skinIII/IV          0.94748907 3.843870e-01  2.335499
```

skinIV	0.95750622	4.157789e-01	2.205062
smoke	0.93659702	8.435969e-01	1.039850
pustular.	0.85717070	6.862402e-01	1.070677
RAPTOR_rs869190	1.24241318	1.044419e+00	1.477942
RAPTOR_rs11658698	1.04722533	9.668499e-01	1.134282
RAPTOR_rs12602885	0.85751628	7.177838e-01	1.024451
ZNF750_rs8074277	1.21540051	5.757513e-01	2.565689
ZNF750_rs11077947	0.97310527	8.936930e-01	1.059574
ZNF750_rs12450046	0.87642458	4.219256e-01	1.820511
TRAF3IP2_rs13190932	1.12051352	5.954741e-01	2.108489
TRAF3IP2_rs13196377	0.98116289	5.792969e-01	1.661809
TRAF3IP2_rs13210247	0.96840684	7.296620e-01	1.285269
TRAF3IP2_rs33980500	0.92346513	7.529845e-01	1.132544

LZS is sigificantly associated with NAPSI and RAPTOR_rs869190GT.

******* NAPSI as dependent variable ***********

glm(formula = NAPSI ~ ., data = CASES_PSO2)

	Estimate	Std. Error	t value	Pr(>|t|)	
sexM	0.058305	0.062414	0.934	0.351369	
FH01	-0.066559	0.060272	-1.104	0.270810	
yob	-0.006773	0.001865	-3.632	0.000360	***
LZS	0.259150	0.080199	3.231	0.001445	**
PASI	0.187939	0.065159	2.884	0.004361	**
skinII	0.488857	0.437483	1.117	0.265179	
skinIII	0.465189	0.441263	1.054	0.293079	
skinIII/IV	-0.059694	0.537567	-0.111	0.911694	
skinIV	0.403482	0.496322	0.813	0.417237	
smoke	0.045947	0.062301	0.738	0.461697	
pustular.	-0.105786	0.132470	-0.799	0.425508	
RAPTOR_rs869190GT	-0.179716	0.110146	-1.632	0.104366	
RAPTOR_rs869190TT	0.078086	0.442484	0.176	0.860106	
RAPTOR_rs11658698CT	-0.056515	0.063433	-0.891	0.374057	
RAPTOR_rs11658698TT	-0.049248	0.109467	-0.450	0.653289	
RAPTOR_rs12602885AA	-0.402558	0.460178	-0.875	0.382760	
RAPTOR_rs12602885AG	0.117560	0.112438	1.046	0.297053	
ZNF750_rs8074277CC	-0.316866	0.494388	-0.641	0.522319	
ZNF750_rs8074277CT	-0.054759	0.072847	-0.752	0.453132	
ZNF750_rs11077947AG	0.029030	0.078823	0.368	0.713051	
ZNF750_rs11077947GG	-0.091140	0.102098	-0.893	0.373131	
ZNF750_rs12450046AA	0.077335	0.435123	0.178	0.859118	
ZNF750_rs12450046AG	NA	NA	NA	NA	
TRAF3IP2_rs13190932AA	0.187147	0.198065	0.945	0.345885	
TRAF3IP2_rs13190932AG	-0.681197	0.374943	-1.817	0.070775	.
TRAF3IP2_rs13196377AA	NA	NA	NA	NA	
TRAF3IP2_rs13196377AG	0.418620	0.312922	1.338	0.182519	
TRAF3IP2_rs13210247AG	0.272073	0.170329	1.597	0.111801	
TRAF3IP2_rs13210247GG	NA	NA	NA	NA	
TRAF3IP2_rs33980500CT	0.024671	0.121800	0.203	0.839691	
TRAF3IP2_rs33980500TT	NA	NA	NA	NA	

	OR	2.5 %	97.5 %
sexM	1.060038e+00	0.9379827	1.197975e+00
FH01	9.356077e-01	0.8313624	1.052924e+00
yob	9.932499e-01	0.9896258	9.968873e-01
LZS	1.295828e+00	1.1073426	1.516397e+00
PASI	1.206760e+00	1.0620810	1.371149e+00
skinII	1.630451e+00	0.6917056	3.843213e+00
skinIII	1.592315e+00	0.6705411	3.781225e+00

```
skinIII/IV           9.420524e-01   0.3284713 2.701797e+00
skinIV               1.497028e+00   0.5659260 3.960046e+00
smoke                1.047019e+00   0.9266678 1.183001e+00
L.kros1k.            8.996170e-01   0.6939034 1.166316e+00
RAPTOR_rs869190GT    8.355072e-01   0.6732770 1.036828e+00
RAPTOR_rs869190TT    1.081215e+00   0.4542229 2.573684e+00
RAPTOR_rs11658698CT  9.450522e-01   0.8345669 1.070164e+00
RAPTOR_rs11658698TT  9.519453e-01   0.7681279 1.179751e+00
RAPTOR_rs12602885AA  6.686077e-01   0.2713108 1.647690e+00
RAPTOR_rs12602885AG  1.124749e+00   0.9022951 1.402047e+00
ZNF750_rs8074277CC   7.284282e-01   0.2764159 1.919599e+00
ZNF750_rs8074277CT   9.467130e-01   0.8207499 1.092008e+00
ZNF750_rs11077947AG  1.029456e+00   0.8820903 1.201441e+00
ZNF750_rs11077947GG  9.128901e-01   0.7473297 1.115128e+00
ZNF750_rs12450046AA  1.080404e+00   0.4604782 2.534913e+00
ZNF750_rs12450046AG            NA          NA           NA
TRAF3IP2_rs13190932AA 1.205804e+00   0.8178692 1.777746e+00
TRAF3IP2_rs13190932AG 5.060110e-01   0.2426656 1.055144e+00
TRAF3IP2_rs13196377AA          NA          NA           NA
TRAF3IP2_rs13196377AG 1.519863e+00   0.8230853 2.806493e+00
TRAF3IP2_rs13210247AG 1.312683e+00   0.9401031 1.832924e+00
TRAF3IP2_rs13210247GG          NA          NA           NA
TRAF3IP2_rs33980500CT 1.024978e+00   0.8073066 1.301340e+00
TRAF3IP2_rs33980500TT          NA          NA           NA
```

per allele:

```
glm(formula = NAPSI ~ ., data = CASES_PSO)

Coefficients:
                      Estimate Std. Error t value Pr(>|t|)
sexM                  0.068397   0.061580   1.111 0.268017
FH01                 -0.064752   0.059199  -1.094 0.275338
yob                  -0.006655   0.001829  -3.639 0.000348 ***
LZS                   0.245142   0.079417   3.087 0.002307 **
PASI                  0.193058   0.064358   3.000 0.003042 **
skinII                0.544372   0.433498   1.256 0.210652
skinIII               0.521111   0.436841   1.193 0.234305
skinIII/IV            0.049235   0.531667   0.093 0.926310
skinIV                0.485872   0.490417   0.991 0.323002
smoke                 0.042173   0.061788   0.683 0.495676
L.kros1k.            -0.115693   0.131415  -0.880 0.379709
RAPTOR_rs869190      -0.154167   0.103244  -1.493 0.136936
RAPTOR_rs11658698    -0.043929   0.047108  -0.933 0.352182
RAPTOR_rs12602885     0.054061   0.105496   0.512 0.608900
ZNF750_rs8074277     -0.182289   0.440408  -0.414 0.679380
ZNF750_rs11077947    -0.045484   0.050115  -0.908 0.365180
ZNF750_rs12450046     0.111158   0.430865   0.258 0.796679
TRAF3IP2_rs13190932 -0.619450   0.370114  -1.674 0.095742 .
TRAF3IP2_rs13196377  0.409036   0.309192   1.323 0.187357
TRAF3IP2_rs13210247  0.260608   0.165828   1.572 0.117618
TRAF3IP2_rs33980500  0.007141   0.120445   0.059 0.952782

                        OR            2.5 %   97.5 %
sexM                1.070790e+00   0.9490464 1.208151e+00
FH01                9.372995e-01   0.8346193 1.052612e+00
yob                 9.933670e-01   0.9898124 9.969344e-01
LZS                 1.277803e+00   1.0936141 1.493013e+00
PASI                1.212953e+00   1.0692091 1.376022e+00
skinII              1.723525e+00   0.7369254 4.030991e+00
skinIII             1.683897e+00   0.7152796 3.964199e+00
```

```
skinIII/IV          1.050467e+00   0.3705323 2.978095e+00
skinIV              1.625591e+00   0.6216804 4.250651e+00
smoke               1.043075e+00   0.9241055 1.177361e+00
L.kros1k.           8.907490e-01   0.6884859 1.152433e+00
RAPTOR_rs869190     8.571289e-01   0.7001080 1.049367e+00
RAPTOR_rs11658698   9.570215e-01   0.8726155 1.049592e+00
RAPTOR_rs12602885   1.055549e+00   0.8583809 1.298005e+00
ZNF750_rs8074277    8.333608e-01   0.3515257 1.975646e+00
ZNF750_rs11077947   9.555352e-01   0.8661408 1.054156e+00
ZNF750_rs12450046   1.117572e+00   0.4803106 2.600330e+00
TRAF3IP2_rs13190932 5.382403e-01   0.2605762 1.111777e+00
TRAF3IP2_rs13196377 1.505367e+00   0.8212168 2.759477e+00
TRAF3IP2_rs13210247 1.297719e+00   0.9376220 1.796113e+00
TRAF3IP2_rs33980500 1.007166e+00   0.7953859 1.275336e+00
```

LZS and PASI are significantly associated with NAPSI

******* Pustular psoriasis as dependent variable ***********

```
glm(formula = L.kros1k. ~ ., data = CASES_PSO2)

                        Estimate Std. Error t value Pr(>|t|)
sexM                   -0.087618   0.033087  -2.648  0.00875 **
FH01                   -0.028885   0.032481  -0.889  0.37494
yob                    -0.001829   0.001029  -1.777  0.07709 .
LZS                    -0.060256   0.044099  -1.366  0.17338
PASI                   -0.052178   0.035619  -1.465  0.14455
NAPSI                  -0.030657   0.038390  -0.799  0.42551
skinII                  0.073910   0.236201   0.313  0.75468
skinIII                 0.109024   0.238091   0.458  0.64752
skinIII/IV             -0.054377   0.289372  -0.188  0.85114
skinIV                  0.203394   0.267241   0.761  0.44752
smoke                   0.085845   0.033020   2.600  0.01004 *
RAPTOR_rs869190GT       0.041144   0.059624   0.690  0.49097
RAPTOR_rs869190TT       0.061268   0.238182   0.257  0.79727
RAPTOR_rs11658698CT    -0.001251   0.034217  -0.037  0.97087
RAPTOR_rs11658698TT    -0.076710   0.058705  -1.307  0.19284
RAPTOR_rs12602885AA    -0.067324   0.248165  -0.271  0.78646
RAPTOR_rs12602885AG    -0.070076   0.060490  -1.158  0.24808
ZNF750_rs8074277CC      0.125753   0.266272   0.472  0.63726
ZNF750_rs8074277CT      0.018998   0.039249   0.484  0.62889
ZNF750_rs11077947AG    -0.006785   0.042445  -0.160  0.87316
ZNF750_rs11077947GG    -0.033260   0.055023  -0.604  0.54623
ZNF750_rs12450046AA    -0.158273   0.233986  -0.676  0.49957
ZNF750_rs12450046AG           NA         NA      NA       NA
TRAF3IP2_rs13190932AA -0.023369   0.106854  -0.219  0.82711
TRAF3IP2_rs13190932AG -0.042490   0.203513  -0.209  0.83483
TRAF3IP2_rs13196377AA        NA         NA      NA       NA
TRAF3IP2_rs13196377AG -0.064513   0.169160  -0.381  0.70334
TRAF3IP2_rs13210247AG -0.011631   0.092285  -0.126  0.89983
TRAF3IP2_rs13210247GG        NA         NA      NA       NA
TRAF3IP2_rs33980500CT  0.144912   0.064753   2.238  0.02635 *
TRAF3IP2_rs33980500TT        NA         NA      NA       NA

                            OR        2.5 %    97.5 %
sexM                    0.9161111 0.8585870    0.9774893
FH01                    0.9715282 0.9116059    1.0353893
yob                     0.9981730 0.9961619    1.0001881
LZS                     0.9415232 0.8635627    1.0265220
PASI                    0.9491596 0.8851564    1.0177906
```

NAPSI	0.9698083	0.8995151	1.0455947
skinII	1.0767104	0.6777108	1.7106195
skinIII	1.1151893	0.6993355	1.7783269
skinIII/IV	0.9470753	0.5371194	1.6699297
skinIV	1.2255550	0.7258667	2.0692301
smoke	1.0896373	1.0213508	1.1624895
RAPTOR_rs869190GT	1.0420022	0.9270788	1.1711718
RAPTOR_rs869190TT	1.0631839	0.6666040	1.6956993
RAPTOR_rs11658698CT	0.9987495	0.9339656	1.0680270
RAPTOR_rs11658698TT	0.9261581	0.8254972	1.0390936
RAPTOR_rs12602885AA	0.9348926	0.5748092	1.5205466
RAPTOR_rs12602885AG	0.9323230	0.8280887	1.0496777
ZNF750_rs8074277CC	1.1340017	0.6729193	1.9110166
ZNF750_rs8074277CT	1.0191801	0.9437180	1.1006763
ZNF750_rs11077947AG	0.9932379	0.9139534	1.0794003
ZNF750_rs11077947GG	0.9672870	0.8683997	1.0774349
ZNF750_rs12450046AA	0.8536170	0.5396274	1.3503057
ZNF750_rs12450046AG	NA	NA	NA
TRAF3IP2_rs13190932AA	0.9769015	0.7923127	1.2044948
TRAF3IP2_rs13190932AG	0.9583996	0.6431555	1.4281613
TRAF3IP2_rs13196377AA	NA	NA	NA
TRAF3IP2_rs13196377AG	0.9375241	0.6729660	1.3060859
TRAF3IP2_rs13210247AG	0.9884362	0.8248896	1.1844084
TRAF3IP2_rs13210247GG	NA	NA	NA
TRAF3IP2_rs33980500CT	1.1559380	1.0181608	1.3123591
TRAF3IP2_rs33980500TT	NA	NA	NA

per allele:

glm(formula = L.kros1k. ~ ., data = CASES_PSO)

Coefficients:

	Estimate	Std. Error	t value	Pr(>|t|)	
sexM	-0.090065	0.032393	-2.780	0.00594	**
FH01	-0.032783	0.031644	-1.036	0.30144	
yob	-0.001706	0.001002	-1.703	0.09004	.
LZS	-0.058714	0.043231	-1.358	0.17593	
PASI	-0.057494	0.034916	-1.647	0.10118	
NAPSI	-0.033037	0.037527	-0.880	0.37971	
skinII	0.071761	0.232500	0.309	0.75791	
skinIII	0.102150	0.234149	0.436	0.66311	
skinIII/IV	-0.042377	0.284102	-0.149	0.88158	
skinIV	0.208449	0.262295	0.795	0.42771	
smoke	0.086999	0.032485	2.678	0.00801	
RAPTOR_rs869190	0.046813	0.055377	0.845	0.39891	
RAPTOR_rs11658698	-0.023163	0.025175	-0.920	0.35863	
RAPTOR_rs12602885	-0.062285	0.056241	-1.107	0.26941	
ZNF750_rs8074277	0.143281	0.235229	0.609	0.54313	
ZNF750_rs11077947	-0.016378	0.026810	-0.611	0.54197	
ZNF750_rs12450046	-0.130931	0.230099	-0.569	0.56997	
TRAF3IP2_rs13190932	-0.049512	0.199117	-0.249	0.80388	
TRAF3IP2_rs13196377	-0.073194	0.165860	-0.441	0.65947	
TRAF3IP2_rs13210247	-0.008649	0.089153	-0.097	0.92281	
TRAF3IP2_rs33980500	0.144931	0.063551	2.281	0.02362	*

	OR	2.5 %	97.5 %
sexM	0.9138718	0.8576538	0.9737748
FH01	0.9677485	0.9095510	1.0296698
yob	0.9982952	0.9963371	1.0002571
LZS	0.9429760	0.8663678	1.0263583
PASI	0.9441277	0.8816792	1.0109993

```
NAPSI                0.9675025 0.8988955    1.0413459
skinII               1.0743981 0.6811788    1.6946085
skinIII              1.1075495 0.6999307    1.7525532
skinIII/IV           0.9585084 0.5492470    1.6727234
skinIV               1.2317663 0.7366522    2.0596534
smoke                1.0908957 1.0236049    1.1626101
RAPTOR_rs869190      1.0479265 0.9401429    1.1680672
RAPTOR_rs11658698    0.9771032 0.9300607    1.0265251
RAPTOR_rs12602885    0.9396153 0.8415458    1.0491134
ZNF750_rs8074277     1.1540539 0.7277785    1.8300079
ZNF750_rs11077947    0.9837557 0.9333967    1.0368316
ZNF750_rs12450046    0.8772783 0.5588264    1.3772026
TRAF3IP2_rs13190932  0.9516938 0.6441818    1.4060023
TRAF3IP2_rs13196377  0.9294208 0.6714788    1.2864487
TRAF3IP2_rs13210247  0.9913883 0.8324472    1.1806765
TRAF3IP2_rs33980500  1.1559602 1.0205824    1.3092955
```

******* PASI as dependent variable (mild/severe) <10>***********

```
glm(formula = PASI ~ ., data = CASES_PSO2)

Deviance Residuals:
    Min       1Q   Median       3Q      Max
-0.6712  -0.3540  -0.1576   0.4755   0.9381

Coefficients: (4 not defined because of singularities)
                        Estimate Std. Error t value Pr(>|t|)
sexM                   0.1570427  0.0662172   2.372  0.01868 *
FH01                   0.0545064  0.0647961   0.841  0.40126
yob                    0.0044449  0.0020442   2.174  0.03088 *
LZS                   -0.0305096  0.0883441  -0.345  0.73020
NAPSI                  0.2166505  0.0751132   2.884  0.00436 **
skinII                 0.2262016  0.4709297   0.480  0.63153
skinIII                0.2505646  0.4747748   0.528  0.59827
skinIII/IV             0.5421980  0.5758867   0.942  0.34761
skinIV                 0.2138627  0.5335654   0.401  0.68899
smoke                  0.1077746  0.0665394   1.620  0.10690
pustular.             -0.2075553  0.1416867  -1.465  0.14455
RAPTOR_rs869190GT     -0.0843323  0.1189084  -0.709  0.47903
RAPTOR_rs869190TT     -0.4862626  0.4738484  -1.026  0.30606
RAPTOR_rs11658698CT   -0.0323401  0.0682053  -0.474  0.63591
RAPTOR_rs11658698TT    0.0476735  0.1175428   0.406  0.68549
RAPTOR_rs12602885AA    0.2974081  0.4945870   0.601  0.54832
RAPTOR_rs12602885AG    0.0210348  0.1210478   0.174  0.86222
ZNF750_rs8074277CC     0.1477964  0.5312607   0.278  0.78115
ZNF750_rs8074277CT    -0.0936975  0.0780399  -1.201  0.23134
ZNF750_rs11077947AG    0.0007293  0.0846597   0.009  0.99314
ZNF750_rs11077947GG    0.1059471  0.1095816   0.967  0.33482
ZNF750_rs12450046AA   -0.3354045  0.4666010  -0.719  0.47310
ZNF750_rs12450046AG           NA         NA      NA       NA
TRAF3IP2_rs13190932AA  0.2160520  0.2125805   1.016  0.31073
TRAF3IP2_rs13190932AG  0.9750537  0.3999211   2.438  0.01566 *
TRAF3IP2_rs13196377AA         NA         NA      NA       NA
TRAF3IP2_rs13196377AG -0.6685707  0.3341097  -2.001  0.04677 *
TRAF3IP2_rs13210247AG -0.3535427  0.1823234  -1.939  0.05393 .
TRAF3IP2_rs13210247GG         NA         NA      NA       NA
TRAF3IP2_rs33980500CT -0.0201474  0.1307786  -0.154  0.87772
TRAF3IP2_rs33980500TT         NA         NA      NA       NA
```

	OR	2.5 %	97.5 %
sexM	1.1700456236	1.027634e+00	1.3321925
FH01	1.0560192014	9.300735e-01	1.1990198
yob	1.0044547543	1.000438e+00	1.0084873
LZS	0.9699511430	8.157390e-01	1.1533165
NAPSI	1.2419099756	1.071898e+00	1.4388866
skinII	1.2538284005	4.981751e-01	3.1556887
skinIII	1.2847506313	5.066288e-01	3.2579757
skinIII/IV	1.7197827630	5.562601e-01	5.3170315
skinIV	1.2384525671	4.352188e-01	3.5241231
smoke	1.1137966254	9.776141e-01	1.2689495
pustular.	0.8125683167	6.155395e-01	1.0726643
RAPTOR_rs869190GT	0.9191257952	7.280478e-01	1.1603526
RAPTOR_rs869190TT	0.6149203127	2.429284e-01	1.5565366
RAPTOR_rs11658698CT	0.9681772220	8.470293e-01	1.1066526
RAPTOR_rs11658698TT	1.0488281938	8.330130e-01	1.3205563
RAPTOR_rs12602885AA	1.3463645804	5.107043e-01	3.5494074
RAPTOR_rs12602885AG	1.0212575731	8.055625e-01	1.2947065
ZNF750_rs8074277CC	1.1592768086	4.092392e-01	3.2839538
ZNF750_rs8074277CT	0.9105581423	7.814118e-01	1.0610488
ZNF750_rs11077947AG	1.0007296022	8.477237e-01	1.1813516
ZNF750_rs11077947GG	1.1117630907	8.968839e-01	1.3781239
ZNF750_rs12450046AA	0.7150487966	2.865261e-01	1.7844616
ZNF750_rs12450046AG	NA	NA	NA
TRAF3IP2_rs13190932AA	1.2411669140	8.182412e-01	1.8826909
TRAF3IP2_rs13190932AG	2.6513096708	1.210729e+00	5.8059587
TRAF3IP2_rs13196377AA	NA	NA	NA
TRAF3IP2_rs13196377AG	0.5124404905	2.662250e-01	0.9863660
TRAF3IP2_rs13210247AG	0.7021960187	4.912069e-01	1.0038118
TRAF3IP2_rs13210247GG	NA	NA	NA
TRAF3IP2_rs33980500CT	0.9800542396	7.584574e-01	1.2663945
TRAF3IP2_rs33980500TT	NA	NA	NA

per allele:

```
glm(formula = PASI ~ ., data = CASES_PSO)
```

Coefficients:

	Estimate	Std. Error	t value	Pr(>|t|)	
sexM	0.1544092	0.0651732	2.369	0.01877	*
FH01	0.0551369	0.0633930	0.870	0.38546	
yob	0.0042946	0.0019968	2.151	0.03269	*
LZS	-0.0269939	0.0869114	-0.311	0.75643	
NAPSI	0.2209058	0.0736410	3.000	0.00304	**
skinII	0.2201310	0.4652590	0.473	0.63663	
skinIII	0.2526911	0.4685922	0.539	0.59030	
skinIII/IV	0.5088197	0.5676057	0.896	0.37109	
skinIV	0.2035734	0.5256746	0.387	0.69897	
smoke	0.1086573	0.0657272	1.653	0.09985	.
pustular.	-0.2303788	0.1399070	-1.647	0.10118	
RAPTOR_rs869190	-0.1072771	0.1107901	-0.968	0.33406	
RAPTOR_rs11658698	0.0004809	0.0504999	0.010	0.99241	
RAPTOR_rs12602885	0.0207468	0.1129122	0.184	0.85440	
ZNF750_rs8074277	0.2852304	0.4708740	0.606	0.54536	
ZNF750_rs11077947	0.0552450	0.0535764	1.031	0.30371	
ZNF750_rs12450046	-0.3833005	0.4601803	-0.833	0.40586	
TRAF3IP2_rs13190932	0.9874854	0.3925427	2.516	0.01266	*
TRAF3IP2_rs13196377	-0.6464257	0.3290417	-1.965	0.05084	.
TRAF3IP2_rs13210247	-0.3403635	0.1768522	-1.925	0.05569	.
TRAF3IP2_rs33980500	-0.0033384	0.1288407	-0.026	0.97935	

	OR	2.5 %	97.5 %
sexM	1.1669683617	1.027031e+00	1.3259728
FH01	1.0566852478	9.332229e-01	1.1964812
yob	1.0043037936	1.000381e+00	1.0082420
LZS	0.9733671503	8.209139e-01	1.1541328
NAPSI	1.2472059246	1.079580e+00	1.4408591
skinII	1.2462400342	5.006942e-01	3.1019219
skinIII	1.2874855447	5.138969e-01	3.2255867
skinIII/IV	1.6633268866	5.468028e-01	5.0596964
skinIV	1.2257750749	4.374776e-01	3.4345179
smoke	1.1147802648	9.800363e-01	1.2680500
L.kros1k.	0.7942326659	6.037522e-01	1.0448087
RAPTOR_rs869190	0.8982767358	7.229453e-01	1.1161302
RAPTOR_rs11658698	1.0004810257	9.061983e-01	1.1045731
RAPTOR_rs12602885	1.0209635571	8.182749e-01	1.2738587
ZNF750_rs8074277	1.3300684331	5.285247e-01	3.3472080
ZNF750_rs11077947	1.0567994594	9.514551e-01	1.1738075
ZNF750_rs12450046	0.6816080348	2.765849e-01	1.6797354
TRAF3IP2_rs13190932	2.6844754650	1.243731e+00	5.7941859
TRAF3IP2_rs13196377	0.5239150511	2.749034e-01	0.9984852
TRAF3IP2_rs13210247	0.7115116724	5.030894e-01	1.0062801
TRAF3IP2_rs33980500	0.9966671725	7.742492e-01	1.2829789

Common variants of ZNF750, RAPTOR and TRAF3IP2 genes and psoriasis risk.
Archives of Dermatological Research.
T. Dębniak, E. Soczawa[2], M. Boer, M. Różewicka-Czabańska, J. Wiśniewska, P. Serrano-Fernandez, A. Mirecka , K. Paszkowska-Szczur, J. Lubinski , L. Krysztoforska, Z. Adamski, R. Maleszka

Department of Genetics and Pathology, International Hereditary Cancer Center, Pomeranian Medical University, Szczecin, Poland
Email:debniak@pum.edu.pl
