# Supplementary material for: Common variants of ZNF750, RPTOR and TRAF3IP2 genes and psoriasis risk
Source: Arch Dermatol Res. 2013 Sep 5;306(3):231–8. doi: 10.1007/s00403-013-1407-9 (PMC3955134; doi:10.1007/s00403-013-1407-9)
Supplement: Supplementary file 2 — Supplementary material 2 (PDF 62 kb) [file 403_2013_1407_MOESM2_ESM.pdf]

Online Resorce 2. Haplotype frequency of examined RAPTOR variants

rs11658698	rs12602885	rs869190	**p-value**	**OR**	**CI 95%**
C	G	G	-	-	-
T	G	G	0.982	0.981	0.798 - 1.21
C	A	T	0.823	0.965	0.761 - 1.23
C	G	T	0.888	0.875	0.496 - 1.54
C	A	G	0.978	0.978	0.464 - 2.06

Haplotypes not frequent enough to allow haplotype analysis were excluded from the table. The reference haplotype corresponds to the most frequent one (CGG).

Common variants of ZNF750, RAPTOR and TRAF3IP2 genes and psoriasis risk.

Archives of Dermatological Research.

T. Dębniak, E. Soczawa[2], M. Boer, M. Różewicka-Czabańska, J. Wiśniewska, P. Serrano-Fernandez, A. Mirecka , K. Paszkowska-Szczur, J. Lubinski , L. Krysztoforska, Z. Adamski, R. Maleszka

Department of Genetics and Pathology, International Hereditary Cancer Center, Pomeranian Medical University, Szczecin, Poland
Email:debniak@pum.edu.pl
